# A simple effective model for STDP: from spike pairs and triplets to rate-encoding plasticity

**DOI:** 10.1186/1471-2202-16-S1-P87

**Published:** 2015-12-18

**Authors:** Rodrigo Echeveste, Claudius Gros

**Affiliations:** 1 Institute for Theoretical Physics, Goethe University Frankfurt, Hessen, 60438, Germany

## 

**Figure 1:**
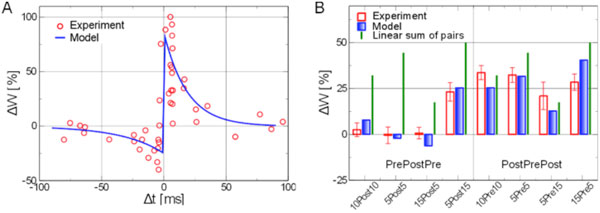
**Model's prediction and comparison to experimental results from hippocampal culture**. **A **. The standard pairwise STDP curve is recovered by the model. Blue lines indicate the model's results and red circles the experimental data [2]. **B **. Triplets, consisting of two pre- and one postsynaptic spike or vice versa, induce a non-linear change in synaptic strength. Blue bars represent the model's results, which follow closely the experimental results [3] presented with red boxes. In green, the linear addition of the contribution of the two composing pairs as from Panel **A**.

In the present work [1] we propose an effective model formulating synaptic potentiation and depression in terms of two interacting traces, representing the fraction of open NMDA receptors and the Ca2+ concentration in the post-synaptic neuron, respectively. These two traces then determine the evolution of the synaptic strength. We first confirm that the standard pairwise STDP curve is obtained for low frequency trains of pairs of pre- and post-synaptic spikes and we then evaluate triplet effects (see Figure 1), comparing the model's results to experimental data from hippocampal culture [2,3]. Finally, we evaluate the model's predictions for spike trains of different frequencies and degrees of correlation, observing that a BCM-like rule for plasticity as a function of the pre-and post-synaptic firing rates is recovered when employing uncorrelated poisson trains of pre- and postsynaptic spikes.

Having a low number of parameters and being composed of only polynomial differential equations, the model is able nonetheless to reproduce key features of LTP and LTD. Moreover, since the parameters of the model are easily related to the dynamical properties of the synapse, we believe the model constitutes a useful tool to study extended neural networks from a dynamical system's point of view.
